# Olfactory Enrichment Influences Adult Neurogenesis Modulating GAD67 and Plasticity-Related Molecules Expression in Newborn Cells of the Olfactory Bulb

**DOI:** 10.1371/journal.pone.0006359

**Published:** 2009-07-23

**Authors:** Serena Bovetti, Alexandra Veyrac, Paolo Peretto, Aldo Fasolo, Silvia De Marchis

**Affiliations:** Department of Animal & Human Biology, University of Torino, Torino, Italy; Institut de la Vision, France

## Abstract

The olfactory bulb (OB) is a highly plastic region of the adult mammalian brain characterized by continuous integration of inhibitory interneurons of the granule (GC) and periglomerular cell (PGC) types. Adult-generated OB interneurons are selected to survive in an experience-dependent way but the mechanisms that mediate the effects of experience on OB neurogenesis are unknown. Here we focus on the new-generated PGC population which is composed by multiple subtypes. Using paradigms of olfactory enrichment and/or deprivation combined to BrdU injections and quantitative confocal immunohistochemical analyses, we studied the effects of olfactory experience on adult-generated PGCs at different survival time and compared PGC to GC modulation. We show that olfactory enrichment similarly influences PGCs and GCs, increasing survival of newborn cells and transiently modulating GAD67 and plasticity-related molecules expression. However, PGC maturation appears to be delayed compared to GCs, reflecting a different temporal dynamic of adult generated olfactory interneuron integration. Moreover, olfactory enrichment or deprivation do not selectively modulate the survival of specific PGC phenotypes, supporting the idea that the integration rate of distinct PGC subtypes is independent from olfactory experience.

## Introduction

Different forms of plasticity, ranging from molecular, synaptic or morphological changes in individual cells to neurogenesis persist in the adult mammalian brain. The dentate gyrus of the hippocampus and the olfactory bulb (OB) represent the two main regions in which new neurons are continuously generated and integrated in adulthood [1], [2]. Neurogenesis in these systems has been demonstrated to be modulated by experience and correlated to learning and memory functions, suggesting that continual addition of new neurons in adult might be crucial for the processing of new informations in response to a complex changing environment [3]. In the adult brain, neuroblasts generated from the subventricular zone (SVZ) migrate tangentially in chains up to the core of the OB, where single cells detach from chains and start to migrate radially [1], [4]. Among the thousands of neuroblasts that every day reach the adult OB, the large majority (about 90%) stops in the granule cell layer (GCL), differentiating into granule cells (GCs), whereas the remaining 10% reaches the glomerular layer (GL) and contributes to periglomerular cells (PGCs) [1]. GCs and PGCs are represented by multiple subtypes of inhibitory interneurons, mainly GABAergic, that modulate the activity of mitral and tufted output neurons by mediating the spatial and temporal coding of olfactory inputs and outputs [5]. Not all the cells that reach the OB survive; about half of newborn GCs [6] and PGCs [7] are eliminated within a time window that for GCs extends from 15 to 45 days after they are born in the SVZ. Studies focused on GCs indicate that the survival of newborn cells is decreased in odor deprived animals [6], [8], whereas higher survival rate occurs in presence of an olfactory enriched environment [9], or when animals are exposed to an olfactory learning experience [10]–[12]. Thus, experience influences the survival of newborn neurons in the OB, and a critical period when their survival is determined in an experience-dependent manner has been identified [13], but the mechanisms underlying this process are still largely unknown.

In this study, we focused on the PGC population which is highly heterogeneous in term of morphological, neurochemical and functional properties [14], [15], and analyzed the effect of olfactory enrichment on adult generated PGCs at different cellular ages following BrdU injection. We show that animals reared in enriched conditions, in parallel to increased survival of 3 weeks old PGCs, display decreased percentage of BrdU/GAD67 double-labelled cells and enhanced proportion of newborn cells expressing the plasticity-related molecules PSA-NCAM and doublecortin (DCX). Similar effects were found on newborn GCs, but at earlier ages compared to PGCs, reflecting a different temporal dynamic of GC integration. Consistently, while GC selection appears to be completed within few weeks after cell birth, continuous olfactory enrichment is necessary to sustain new-generated PGC survival at 6 weeks, and its removal during late phase of PGC maturation (3–6 weeks) cancel the pro-survival effect. Finally, by using paradigms of olfactory enrichment or deprivation we show that olfactory experience does not influence the recruitment of specific PGC subtypes.

## Results

### Continuous olfactory enrichment is critical to sustain survival of adult-born periglomerular cells

**Figure 1:**
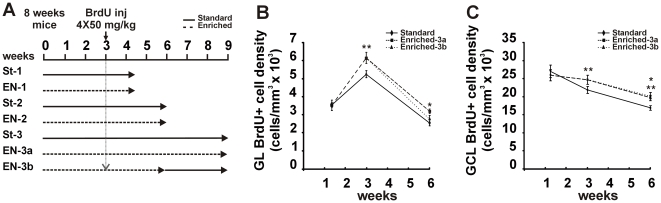
BrdU-positive cell density increases in the GL and GCL of mice reared in enriched olfactory environment. *A,* Experimental procedure. Adult mice were injected with BrdU 3 weeks after initial treatment (four injections, 4-h interval, 50 mg/kg, i.p.). The quantification of neurogenesis (survival of BrdU-labelled cells) was done by counting BrdU-labelled cells in the GL and GCL at 10, 21 and 42 days post-BrdU injection (p.i.). *B,* Mean number of BrdU-IR cells per millimetres cubed in the GL of standard and enriched mice at 10, 21 and 42 days p.i.. *C,* Mean number of BrdU-IR cells per millimetres cubed in the GCL of standard and enriched mice at 10, 21 and 42 days p.i.. Error bars indicate SEM. * p<0.05, ** p<0.01. St-1: standard at 10 days p.i.; St-2: standard at 21 days p.i.; St-3: standard at 42 days p.i.; EN-1: enriched at 10 days p.i.; EN-2: enriched at 21 days p.i.; EN-3a: enriched at 42 days p.i; EN-3b: animals enriched 21 days p.i. and returned to standard housing conditions for 21 more days.

Integration of adult-born interneurons into existing olfactory circuits is modulated by olfactory enrichment, learning or deprivation [8]–[11], [16]. However, most of the studies have been focused on granule cell (GC) integration, whereas few data are available on PGCs which incorporate into the GL. We initially investigated whether olfactory enrichment influences survival of new-generated PGCs at different time points. To this aim, BrdU injections were performed after 3 weeks of initial sensory enrichment and animals were maintained in enriched conditions for additional 10, 21 or 42 days (EN-1, EN-2 and EN-3a respectively; Fig. 1A). An experimental group represented by animals maintained in enriched conditions for 21 days after BrdU injection and returned to standard housing for 21 more days (EN-3b) was added to the long-survival time point (Fig. 1A).

Consistently with the population dynamics of adult generated PG interneurons [17], the density of BrdU-positive cells in the GL increases during the first 3 weeks after injection, declining between 3 and 6 weeks (Fig. 1B). A two-way ANOVA revealed a significant effect of both time and enrichment on BrdU-positive cell density in the GL (time: F_(2,31)_ = 122.463, p<0.001; enrichment: F_(1,31)_ = 5.969, p<0.05). While no difference between standard and enriched animals has been detected in 10-days old BrdU-positive cells (*t-*test p = 0.47, St-1 vs EN-1; 5 animals per group; Fig. 1B), olfactory enrichment induces an increase in the density of 3 weeks old new-generated PGCs by 17% (*t-*test p<0.01, St-2 vs EN-2; 7 animals per group; Fig. 1B). Interestingly, at longer survival time, increased BrdU density by 25% occurs in mice constantly maintained in olfactory enriched environment (One-way ANOVA, F_(2,18)_ = 5.021, p<0.05; Tukey post hoc p<0.05, St-3 vs EN-3a; 7 animals per group; Fig. 1B), whereas mice returned to standard housing for the last 21 days (EN-3b) do not show a significant difference in the number of BrdU-positive PGCs (Tukey post hoc p = 0.6, St-3 vs EN-3b; 7 animals per group; Fig. 1B).

In parallel, we also investigated the time course of GC survival in the different experimental conditions (Fig. 1C). In control condition, the density of BrdU-positive GCs progressively declines starting from 10 days survival, indicating differences in the temporal dynamic of newborn cell selection between PGCs and GCs. As for PGCs, a two-way ANOVA showed a significant effect of both time and enrichment on BrdU-positive cell density in the GCL (time: F_(2,31)_ = 32.261, p<0.001; enrichment: F_(1,31)_ = 4.210, p<0.05). No difference between standard and enriched animals has been detected in 10-days old BrdU-positive cells (*t-*test p = 0.32, St-1 vs EN-1; 5 animals per group; Fig. 1C) while olfactory enrichment induces an increase in the density of 3 weeks old new-generated GCs (*t-*test p<0.01, St-2 vs EN-2; 7 animals per group; Fig. 1C). Interestingly, differently to PGCs, increased BrdU-positive cell density is maintained at longer survival time in both EN-3a and EN-3b compared to control (One-way ANOVA, F_(2,18)_ = 8.181, p<0.01; Tukey post hoc p<0.05, St-3 vs EN-3a; p<0.01 St-3 vs EN-3b; 7 animals per group; Fig. 1C).

### Olfactory enrichment modulates GAD67 expression in newborn olfactory interneurons

**Figure 2:**
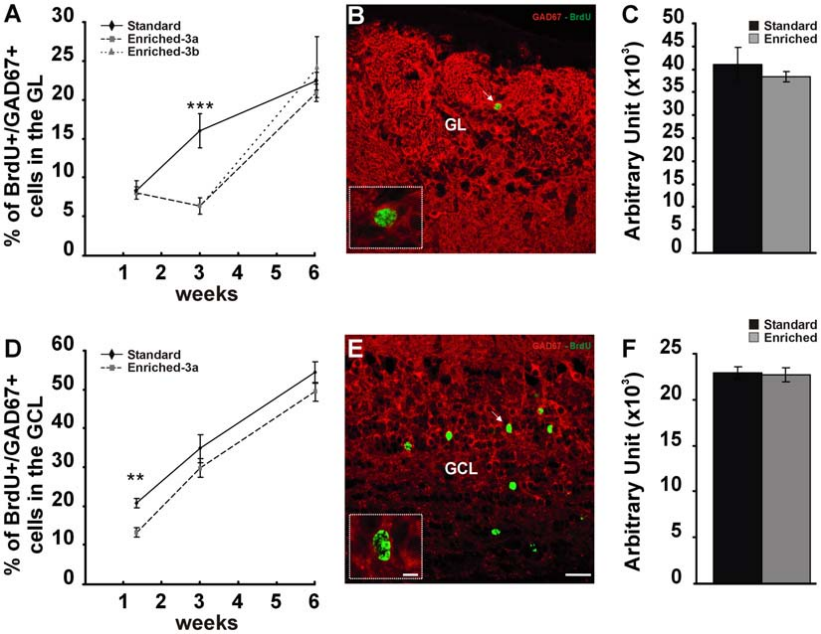
Olfactory enrichment modulates GAD67 expression in new-generated olfactory interneurons. *A,D* Quantification of BrdU-positive PGCs *(A)* and GCs *(D)* double-labelled for GAD67 among the total number of BrdU-positive cells counted in the GL and GCL at 10, 21 and 42 days survival in standard and enriched groups. *B,E* Confocal analysis of olfactory bulb GL *(B)* and GCL *(E)* stained in green for BrdU and red for GAD67 in standard conditions. The arrow shows a BrdU-positive neuron expressing GAD67 (higher magnification in inset; single confocal plane). *C,F* signal intensity of GAD67 expression in the GL *(C)* and GCL *(F)* of standard and olfactory enriched mice. Error bars indicate SEM. ** p<0.01, *** p<0.001. Scale bar in E corresponds to 25 µm and applies to B. Scale bar in insets corresponds to 7 µm. GL: glomerular layer; GCL: granule cell layer.

Olfactory adult-generated cells are inhibitory interneurons, predominantly GABAergic. GABA is synthesized by either GAD65 or GAD67 and about 32% of all PGCs in the GL are GAD67-immunopositive [14]. We investigated GAD67 expression in newborn PGCs in standard and enriched conditions at different survival time (Fig. 2A,B). A two-way ANOVA revealed a significant effect of both time and enrichment on the percentage of BrdU/GAD67 double-positive cells in the GL (time: F_(2,20)_ = 53.745, p<0.001; enrichment: F_(1,20)_ = 10.695, p<0.01). Indeed, in standard conditions the percentage of BrdU/GAD67 PGCs increases with time supporting a progressive maturation of the GABAergic phenotype within several weeks after birth (Fig. 2A,B). Newborn PGCs immunopositive for GAD67 increase from 8.6±0.9% (10 days p.i.; n = 242 out of 3 animals) to 16±0.7% (21 days p.i.; n = 533 out of 6 animals) and to 22.5±1.3% at prolonged survival time (42 days p.i.; n = 257 out of 4 animals). Interestingly, the temporal profile of GAD67 expression in newborn cells is different in odor enriched versus standard mice. Although no significant difference between standard and enriched animals resulted in the percentage of BrdU/GAD67 positive PGCs at 10 days survival (*t-*test p = 0.65, St-1 vs EN-1; n = 242 for St-1 and n = 206 for EN-1; 3 animals per group; Fig. 2A), at 21 days, odor enriched mice show a percentage of double-labelled PGCs which is 2.5-fold less compared to standard (*t-*test p<0.01, St-2 vs EN-2; n = 533 for St-2 and n = 605 for EN-2, 6 animals per group; Fig. 2A). At 42 days survival, independently from maintenance of continuous olfactory enrichment, no significant difference between standard and enriched animals was observed (One-way ANOVA, F_(2,9)_ = 0.398, p = 0.683; n = 257 for St-3, n = 248 for EN-3a and n = 243 for EN-3b, 4 animals per group; Fig. 2A).

To understand whether this effect is specific on new-generated cells or concerns the entire PGC population we evaluated GAD67 expression in the whole GL at 21 days survival. GAD67 immunoreactivity is widely distributed into the GL and the antibody to GAD67 stains both dendrites and cell bodies (Fig. 2B) hampering the counting of all GAD67 positive neurons in this layer. Therefore, GAD67 signal intensity in the GL was measured by densitometry in both standard and enriched groups. The GL of mice reared in olfactory enriched environment does not show any significant difference in the intensity of GAD67 immunostaining compared to control group (*t-*test, p = 0.532, St-2 vs EN-2, Fig. 2C) indicating that the modulation in GAD67 expression likely occurs selectively on newborn cells.

In order to asses whether olfactory enrichment also modulates GAD67 expression in newborn GCs we extended our analysis to the GCL (Fig. 2D–F) where about 51% of all interneurons express GAD67 [14]. Similarly to PGCs, a two-way ANOVA revealed a significant effect of both time and enrichment on the percentage of BrdU/GAD67 labelled cells in the GCL (time: F_(2,20)_ = 60.756, p<0.001; enrichment: F_(1,20)_ = 5.252, p<0.05). In standard conditions, double-labelled cells progressively increase with time rising from 20.3±1.2% (10 days p.i.; n = 987 out of 4 animals) to 34.9±3.6% (21 days p.i.; n = 1040 out of 6 animals) and to 54.3±2.6% at prolonged survival time (42 days p.i.; n = 505 out of 3 animals). As for PGCs olfactory enrichment induces a decrease in the percentage of newborn GCs labelled for GAD67, however the effect resulted statistically significant at 10 days survival (*t-*test p<0.01, St-1 vs EN-1; n = 987 for St-1 and n = 917 for EN-1, 4 animals per group; Fig. 2D), while no difference has been detected at 21 and 42 days (*t-*test p = 0.28, St-2 vs EN-2; n = 1040 for St-2 and n = 1038 for EN-2, 6 animals per group; *t-*test p = 0.24, St-3 vs EN-3a; n = 505 for St-3 and n = 503 for EN-3a; 3 animals per group Fig. 2D). GAD67 expression was also evaluated by densitometry in the whole GCL of standard (St-1) and enriched (EN-1) mice at short survival time (10 days post-BrdU injection; Fig. 2F). Similarly to GL, no difference was observed (*t-*test p = 0.838, St-1 vs EN-1), further supporting the modulation of GAD67 induced by olfactory enrichment selectively targets newborn cells.

### Olfactory enrichment modulates PSA-NCAM and DCX expression

**Figure 3:**
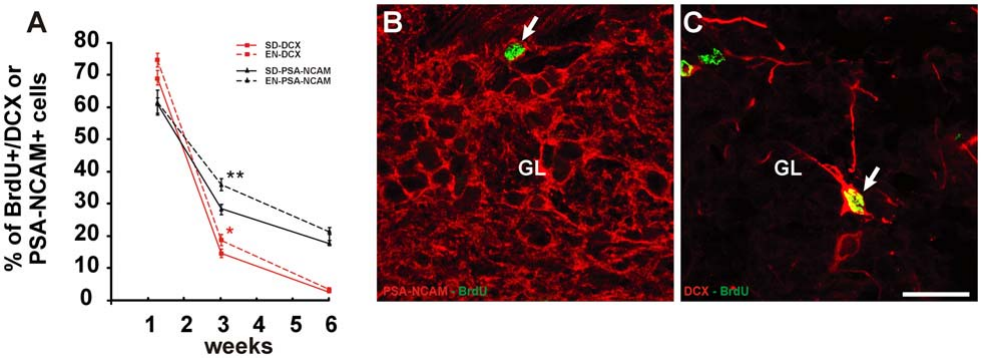
Olfactory enrichment modulates DCX and PSA-NCAM expression in new-generated olfactory interneurons. *A,* Quantification of BrdU-positive PGCs double-labelled for DCX or PSA-NCAM among the total number of BrdU-positive cells counted in the GL at 10, 21 and 42 days survival in standard and enriched groups. Error bars indicate SEM. * p<0.05, ** p<0.01. *B,* Confocal analysis of olfactory bulb GL stained in green for BrdU and red for PSA-NCAM. *C,* Confocal analysis of olfactory bulb GL stained in green for BrdU and red for DCX. The arrows shows BrdU-positive neuron expressing PSA-NCAM (B) or DCX (C). Scale bar in C corresponds to 25 µm and applies to B. GL: glomerular layer.

The modulation in GAD67 expression during newborn cell maturation suggests a possible retard in the maturation process. It has been shown previously that during differentiation, newly generated cells in the OB down-regulate PSA-NCAM and DCX [18], [19]. We thus evaluated the expression of these molecules in newborn olfactory interneurons by immunohistochemistry (Fig. 3). While DCX labelling in the GL is restricted to a few cells (Fig. 3C) representing immature interneurons, PSA-NCAM signal is widely distributed (Fig. 3B) and labels a greater number of cells and processes in accordance to PSA-NCAM expression also in mature neurons undergoing synaptic plasticity [18], [20], [21]. In the GL, a two-way ANOVA showed a significant effect of time and enrichment on the percentage of both BrdU/PSA-NCAM (time: F_(2,26)_ = 173.39, p<0.001; enrichment: F_(1,26)_ = 4.524, p<0.05) and BrdU/DCX positive cells (time: F_(2,26)_ = 1011.523, p<0.001; enrichment: F_(1,26)_ = 6.783, p = 0.01). In control groups the percentage of BrdU/PSA-NCAM double-labelled cells progressively decreases from 62±3.5% (10 days p.i.; n = 345 out of 5 animals) to 28.5±1.4% (21 days p.i.; n = 408 out of 6 animals) to 18±2.3% (42 days p.i.; n = 132 out of 4 animals) whereas the percentage of BrdU/DCX shows a more drastic reduction from 69±2.2% (10 days p.i.; n = 608 out of 5 animals) to 14.6±1.4% (21 days p.i.; n = 580 out of 6 animals) to 2.6±0.3% (42 days p.i.; n = 419 out of 5 animals) (Fig. 3A). Consistent to our hypothesis , at 21 days survival enriched mice show an increase in percentage of BrdU/PSA-NCAM double-labelled cells from 28.5±1.4% to 36±1.8% (*t-*test p<0.01, St-2 vs EN-2; n = 408 out of 6 animals for St-2 and n = 430 out of 7 animals for EN-2; Fig. 3A) paralleled by a statistically significant increase (from 14.6±1.4%, St-2 to 19±1.7%, EN-2) in the percentage of BrdU/DCX double-labelled cells (*t-*test p<0.05, St-2 vs EN-2; n = 580 for St-2 and n = 602 for EN-2, 6 animals per group; Fig. 3A). Thus, the down-regulation of PSA-NCAM and DCX occurs more slowly in newborn PGCs born in an enriched environment. After 42 days survival in continuous enriched conditions, values of both BrdU/PSA-NCAM and BrdU/DCX return similar to control indicating that the delay in PSA-NCAM and DCX down regulation observed in 3 weeks old newborn PGCs is rescued at longer survival time (Fig. 3A).

It is noteworthy that similar effects occur in GCs. The percentage of newborn GCs double-labelled for PSA-NCAM and DCX has been evaluated at 10 and 21 days post BrdU injection. Two-way ANOVA analysis showed a significant effect of time and enrichment on the percentage of both BrdU/PSA-NCAM (time: F_(1,9)_ = 628.685, p<0.001; enrichment: F_(1,9)_ = 6.531, p<0.05) and BrdU/DCX positive cells (time: F_(1,14)_ = 6808.608, p<0.001; enrichment: F_(1,14)_ = 6.230, p<0.05) in the GCL. Interestingly, similarly to GAD67 results, the effect of olfactory enrichment on PSA-NCAM and DCX expression in newborn GCs appears earlier compared to PGCs. A statistically significant increase in the percentage of BrdU cells double-labelled for PSA-NCAM occurs at 10 days survival (from 76.2±0.6% in St-1 to 84±1.5% in EN-1; *t-*test p<0.01, St-1 vs EN-1; n = 363 for St-1 and n = 312 for EN-1, 3 animals per group) and a trend for DCX (from 92±1% in St-1 to 95±0.8%, in EN-1; *t-*test p = 0.06 St-1 vs EN-1; n = 2186 for St-1 and n = 2301 for EN-1; 5 animals per group). In contrast, no effect has been detected at 21 days for PSA-NCAM (from 26.7±2.3% in St-2 to 29.6±2.4% in EN-2; *t-*test p = 0.22, St-2 vs EN-2; n = 331 out of 3 animals for St-2 and n = 817 out of 4 animals for EN-2) and DCX (from 12±1.3% in St-2 to 15±0.5%, in EN-2; *t-*test p = 0.063 St-2 vs EN-2; n = 1503 for St-2 and n = 1520 for EN-2; 4 animals per group).

### Integration rate of distinct periglomerular cell subtypes is independent from olfactory experience

**Figure 4:**
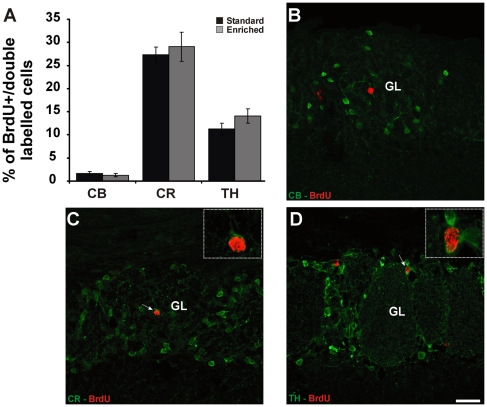
Olfactory enrichment does not alter the relative proportion of new-generated PGCs expressing CB, CR and TH. *A,* Quantification of BrdU-positive PGCs double-labelled for CB, CR and TH among the total number of BrdU-positive cells counted in the GL at 21 days survival in standard and enriched groups. Error bars indicate SEM. *(B–D)* Confocal analysis of olfactory bulb GL stained in red for BrdU and green for CB (B), CR (C) and TH (D) in standard mice. The arrows in C and D show a BrdU-positive neuron expressing CR (higher magnification in inset in C; single confocal plane) and TH (higher magnification in inset in D; single confocal plane). Scale bar in D corresponds to 25 µm in D, B and C and to 10 µm in insets in C and D. GL: glomerular layer.

The mouse OB contains three main non-overlapping populations of PGCs, characterized by expression of calbindin (CB), calretinin (CR) and tyrosine hydroxylase (TH) [14]; we thus investigated whether olfactory enrichment selectively modulates the expression of these molecules and/or survival of specific PGC subtypes. According to BrdU and GAD67 results, the percentages of PGCs double-labelled for BrdU and CB, CR or TH, were evaluated 21 days following BrdU injection on animals reared in standard or enriched conditions. According to previous report [22], standard housed mice show few BrdU positive cells expressing CB (1.63±0.41; % of BrdU/CB double positive cells±SEM; n = 1694 out of 6 animals; Fig. 4A,B), whereas CR and TH are expressed respectively in the 27.36±1.66% (n = 1297 out of 5 animals; Fig. 4A,C) and 11±1.2% of new-generated cells (n = 1287 out of 6 animals, Fig. 4A,D). Mice reared in enriched environment, do not show any statistically significant difference in the relative proportion of double-labelled cells compared to standard group (CB: *t-*test, p = 0.532, St-2 vs EN-2, n = 1694 for St-2 and n = 1567 for EN-2, 6 animals per group; CR: *t-* test, p = 0.645, St-2 vs EN-2, n = 1297 out of 5 animals for St-2 and n = 1555 out of 6 animals for EN-2; TH: *t-* test, p = 0.194, St-2 vs EN-2, n = 1287 for St-2 and n = 1564 for EN-2, 6 animals per group). Thus, broad enhancement of olfactory activity induced by olfactory enrichment does modulate neither CB, CR and TH expression nor selective survival of specific new-generated subtypes.

**Figure 5:**
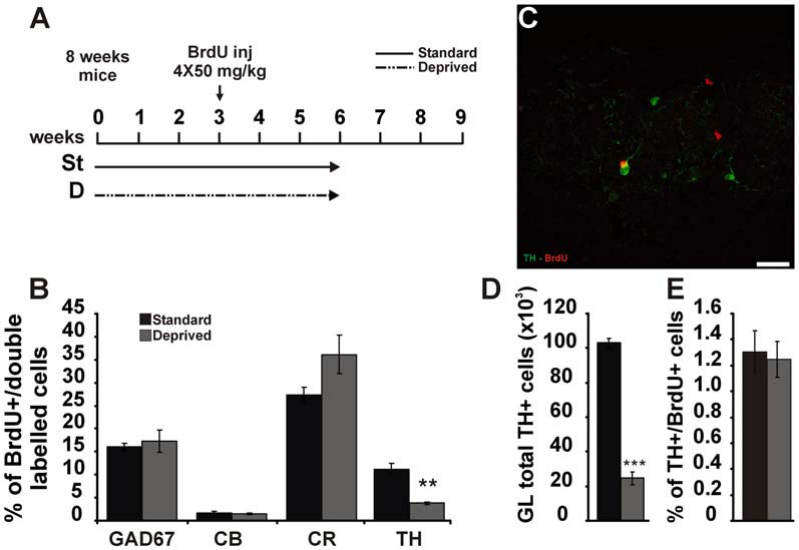
Olfactory deprivation does not alter the relative proportion of new-generated PGCs expressing GAD67, CB and CR whereas it regulates TH expression in both new-generated and resident PGCs. *A,* Experimental procedure. Adult mice were injected with BrdU 3 weeks after unilateral naris closure (four injections, 4-h interval, 50 mg/kg, i.p.). The quantification of neurogenesis (survival of BrdU-labelled cells) was done by counting BrdU-labelled cells in the GL at 21 days post-BrdU injection. *B,* Quantification of BrdU-positive PGCs double-labelled for GAD67, CB, CR and TH among the total number of BrdU-positive cells counted in the GL at 21 days survival in standard and deprived groups. Error bars indicate SEM. ** p<0.01. *C,* Confocal analysis of olfactory bulb GL stained in red for BrdU and green for TH, 21 days p.i. *D,* Total number of TH-positive cells in the GL of standard and deprived animals 42 days after unilateral naris closure. *E,* Quantification of TH-positive PGCs double-labelled for BrdU among the total number of TH-positive cells counted in the GL in standard and deprived groups 42 days after unilateral naris closure. Error bars indicate SEM. *** p<0.001. Scale bar in C corresponds to 25 µm.

We next sought to investigate whether an opposite paradigm of olfactory manipulation represented by olfactory deprivation, which is known to decrease the survival rate of new-generated cells in the OB [23], [24] could affect GAD67, CB, CR, TH expression and survival of specific PGC subtypes. Similarly to the enrichment protocol, BrdU injections were performed after 3 weeks of initial sensory deprivation and the animals were then maintained in the same conditions for 3 more weeks (Fig. 5A). According to previous reports, we observed a strong decrease in BrdU cell density in the GL of OB unilateral to naris occlusion (not shown). No differences were observed in the percentage of BrdU-positive cells double positive for GAD67 (*t-* test, p = 0.538, standard vs deprived, n = 533 out of 6 animals for standard and n = 256 out of 4 animals for deprived; Fig. 5B), suggesting that contrary to olfactory enrichment, odor deprivation does not influence GAD67 expression in new-generated cells.

Moreover, no significant difference has been detected in the percentage of PGCs double-positive for BrdU and CB or CR (CB: *t-* test, p = 0.600, standard vs deprived, n = 1694 out of 6 animals for standard and n = 683 out of 4 animals for deprived; CR: *t-* test, p = 0.076, standard vs deprived, n = 1297 out of 5 animals for standard and n = 675 out of 4 animals for deprived; Fig. 5B). Conversely, odor deprivation resulted in a 3-fold decrease in the percentage of BrdU/TH double-labelled cells compared to control (*t-* test, p = 0.01, standard vs deprived; n = 1287 out of 6 animals for standard and n = 709 out of 4 animals for deprived; Fig. 5B). Sensory activity is known to mediate the regulation of TH expression into the rodent OB, without specifically increasing dopaminergic neuron cell death [25], [26]. Accordingly, both densitometric analysis of TH immunostaining (35×10^3^ vs 7×10^3^ Arbitrary Unit; *t-* test, p<0.001, standard vs deprived) and evaluation of the total number of TH positive PGCs (*t-* test, p<0.001, standard vs deprived; Fig. 5D), show a sharp reduction of TH expression in olfactory deprived animals. To assess whether similarly to the resident cell population the decrease in the percentage of BrdU/TH double-labelled cells reflects a down-regulation of TH expression in newborn elements rather than a selective death of new-generated dopaminergic cells, we evaluated the percentage of TH/BrdU double positive PGCs in standard and deprived mice (Fig. 5E). The proportion of BrdU-positive cells among the TH population is similar in standard and deprived conditions (*t-* test, p = 0.811, standard vs deprived; Fig. 5E) supporting that odor deprivation acts down-regulating TH expression on both pre-existing and newly integrated PGCs.

## Discussion

### PGCs and GCs follow a different temporal dynamic in experience-dependent cell selection

Several studies have shown that among the thousands of newborn olfactory interneurons daily generated during adult life, over half are lost in few weeks after birth [6], [17], and their survival has been shown to be critically regulated by sensory input [8]–[11], [13]. Accordingly, we show that similarly to GCs [9], olfactory enrichment enhances survival of 3 weeks old PGCs. However, our results indicate that at longer survival time (6 weeks) the two populations behave differently: mice enriched for 3 weeks after BrdU injection and placed back to standard conditions for 3 more weeks retain enhanced survival of GCs, whereas the effect is not long-lasting on PGCs. Noteworthy, continuous olfactory enrichment for the whole survival period after BrdU injection (6 weeks) sustains higher survival rate of PGCs, having no effect on GC survival. These results are consistent with findings by Yamaguchi and Mori [13], and more recently by Mouret and colleagues [11], that identified a “critical period” of time for newborn GCs (corresponding to 14–28/30 days after cell birth), when GCs exhibit enhanced synaptic plasticity and their survival is influenced by olfactory experience. Indeed, olfactory deprivation, a procedure that strongly affects newborn GC survival in the “critical period”, produces no effect if performed during a period lasting from 28 to 56 days after cell birth [13]. Our results support that once out from the “critical period”, survival of newborn GCs is no longer susceptible to olfactory experience. In contrast with these results, previous reports by Rochefort and others [27] indicated that increased GC survival following olfactory enrichment is transitory and returns to basal level once animals are placed back to standard conditions, suggesting that GC survival can still be modulated by sensory experience. However, the study performed by Rochefort was based on a different time course (keeping animals in standard housing conditions for a longer period of time) and sample size.

We hypothesize that similarly to GCs the integration of PGCs into olfactory circuits undergoes a “critical period”, during which their fate between survival and death is regulated by sensory experience. The selection of adult born olfactory interneurons has been suggested to depend on their maturation stage [6] and based on morphological and electrophysiological analysis PGCs differentiate more slowly than GCs [7], [28], [29]. Consistently, as shown by our time course analysis in standard conditions, adult generated PGCs and GCs follow a different temporal dynamic in cell selection: while the density of BrdU-immunopositive GCs declines from 10 days to 6 weeks post injection, the density of BrdU-positive cells in the GL increases from 10 days to 3 weeks after injection, declining at 6 weeks. According to the observed sensibility of newborn PGCs to sensory experience at longer survival time, their “critical period” appears to be either shifted or prolonged compared to GCs, possibly as a consequence of their different maturation time course.

### Olfactory enrichment modulates GAD67 expression in new-generated olfactory interneurons and enhances expression of cell plasticity-related molecules

The majority of SVZ-progenitors mature into GABAergic interneurons in the olfactory bulb [14], [30]. GABA is synthesized by two glutamic acid decarboxylases: GAD65, concentrated in axon terminals and bound to synaptic vesicles, and GAD67 that has been found throughout the cell [31], [32]. Between the two isoforms GAD67 is responsible for over 90% of basal GABA synthesis in the brain and its expression has been suggested to be regulated by activity [33], [34]. Our results reveal that GAD67 expression in periglomerular and granule cells correlates with their maturation process. Indeed, the percentage of new-generated cells immunopositive for GAD67 progressively increases while maturation proceeds reaching 22.5% of PGCs and 54.3% of GCs 42 days after cell birth. These values are consistent to the estimated proportion of GAD67 immunopositive cells on the overall neuronal population in the GL and GCL [14].

We show that olfactory enrichment, besides influencing survival of newborn interneurons, also modulates GAD67 expression in these cells. The most striking effect was observed at 3 weeks post-BrdU injection in the GL, where the percentage of BrdU/GAD67 double-positive PGCs is drastically lowered in enriched compared to control animals, returning to control value at 6 weeks. We thus infer that the observed transient reduction reflects a modulation of GAD67 expression in newborn PGCs. Accordingly, in other sensory systems, the GABAergic network has been demonstrated to be dynamically modulated by external inputs [35], [36]. Interestingly, a reduction by 35% was also observed in the percentage of double-labelled BrdU/GAD67 cells in the GCL of enriched mice, indicating that olfactory enrichment influences GAD67 expression in both populations of olfactory interneurons. However, the modulation of GAD67 expression in GCs occurs earlier (at 10 days post-BrdU injection) compared to PGCs, coherently to the different maturation profile of the two populations.

Based on these results we hypothesize that the decreased percentage of BrdU/GAD67 double-labelled cells corresponds to a delayed expression of GAD67 which, in turn, might reflect a retard in the maturation of newborn OB interneurons. We thus analyzed PSA-NCAM and DCX, two cell plasticity-related molecules known to be expressed by neuroblasts and down-regulated once cells become mature [18], [19]. In standard conditions, the percentage of PGCs double-labelled for BrdU and DCX sharply decreases from 10 to 21 days post-BrdU injection reaching almost undetectable levels at 42 days. Down-regulation of PSA-NCAM in newborn PGCs appears to be slower and levels of PSA-NCAM expression remain higher at both 21 and 42 days post-BrdU injection, compared to DCX. These findings are consistent to data obtained in the hippocampus showing that PSA-NCAM expression in newborn cells persists longer compared to DCX [37].

Our results show that olfactory enrichment modulates both PSA-NCAM and DCX expression in adult born PGCs, enhancing the percentage of 3 weeks old BrdU/PSA-NCAM or DCX double-labelled cells. Similarly, newborn GCs display higher percentage of PSA-NCAM labelled cells; however, according to GCs temporal dynamic and GAD67 regulation, PSA-NCAM up-regulation in enriched animals was detected earlier, on 10 days old BrdU-positive cells. A trend to increase in the percentage of BrdU/DCX double-positive GCs was also observed, but the values did not reach a statistically significant difference. In general, both in the GL and GCL, the effect of olfactory enrichment is more evident on PSA-NCAM modulation. This is possibly a consequence of different temporal dynamics in PSA-NCAM and DCX expression in newborn cells and/or of the wider involvement of PSA-NCAM in structural plasticity processes [18].

No difference in DCX and PSA-NCAM expression has been detected in 42 days old PGCs suggesting that olfactory enrichment enhances newborn PGCs plasticity possibly delaying their maturation that is however reached within 6 weeks after cell birth.

### Environmental inputs influence survival of adult-generated OB cells without altering the balance between specific interneuron populations

Periglomerular and granule cells are subdivided into several subtypes, differing in their functions within the OB circuit [38]. The best described are three major classes of PGCs, characterized by expression of calbindin (CB), calretinin (CR) or tyrosine hydroxylase (TH) [5]. Among these subtypes, TH- and CB-positive cells are largely GABAergic (78% and 65% of TH- and CB-positive cells respectively) with preferential use of GAD67, whereas only 14% of CR-positive cells co-express enzymes for GABA synthesis [14].

Specific adult-born interneuron phenotypes are replaced at different rates [30] and their survival or the expression of selective neurochemical markers could be differently modulated in response to olfactory enrichment. Our results show that increased 3-weeks old PGC survival in enriched conditions does not preferentially modulate or recruit a specific cell phenotype, supporting the emerging view of OB interneuron diversity mainly determined by cell autonomous mechanisms [22], [39].

In addition to olfactory enrichment we analyzed newborn cell survival following olfactory deprivation, 3 weeks after BrdU injection (6 weeks after initial deprivation). According to previous work [8], odor deprivation strongly affects adult-born PGC survival. However, also in this case we did not observe a preferential selection on specific PGC phenotypes or a modulation of the percentage of newborn cells expressing selective PGC neurochemical markers, with the exception of TH.

It is noteworthy that olfactory enrichment and deprivation do not act on newborn cells simply as opposite paradigms, but exert distinct regulatory effects on specific neurotransmitter systems. We show that while olfactory deprivation induces a strong reduction in the expression of TH, the rate–limiting enzyme in the dopamine biosynthetic pathway, in both resident [26] and newborn PGCs (this study), olfactory enrichment has no effect. TH expression in PGCs depends on glutamate release by olfactory receptor terminals [40], and reduced synaptic activity following olfactory deprivation is responsible for TH down-regulation [26]. However, olfactory enrichment, a condition of enhanced GL activation, does not result in TH up-regulation. On the other hand, olfactory deprivation has no effect on GAD67 expression modulation on newborn PGCs, as previously demonstrated for resident GCs [41]. Although we can not exclude that following different protocols of olfactory deprivation (i.e. analysis of cells at different survival time) an effect could be found, these findings straighten the specific role played by olfactory enrichment on GAD67 expression modulation and its possible implication in plasticity mechanisms.

### Concluding remarks

In the OB, newborn neurons integrate in pre-existing circuits, where they play a role in brain plasticity and underlie some forms of olfactory learning and memory. While cell autonomous mechanisms appear to govern much of the final identity of new-generated cells, we show that sensory enrichment can modulate survival and enhance plasticity of adult-generated olfactory interneurons likely retarding their maturation. The enhancement of cell plasticity might represent a strategy allowing the establishment of fine-tuned neuronal circuits in response to increased sensory inputs; the formation of proper connections will possibly allow augmented survival of newborn cells according to an activity-dependent selection process.

## Materials and Methods

### Animals and housing conditions

Experiments were performed on 59 male C57BL/6J strain mice (8 weeks; Charles River, Calco, Italy; Charles River). Animals were housed under a 12 h light: dark cycle in an environmentally controlled room. All experimental procedures were in accordance with the European Communities Council Directive of 24 November 1986 (86 609 EEC), the Italian law for care and use of experimental animals (DL116 92) and approved by the Italian Ministry of Health and the Bioethical Committee of the University of Turin.

### Olfactory deprivation and enrichment

On their arrival in the laboratory, mice were held in standard laboratory cages in groups of 5–7 and randomly assigned to nine experimental groups (Fig. 1A and 5A). The enriched groups (Fig. 1A) consisted of animals housed in an odor-exposure environment for 31 (EN-1; n = 5), 42 (EN-2; n = 7) and 63 (EN-3a; n = 7) days. A group of animals maintained in enriched conditions for 42 days before returning in standard housing for 21 more days (EN-3b; n = 7) was also performed. Odor-enriched mice were daily exposed for 24 hr to different aromatic fragrances that were placed in a tea ball hanging from the acrylic filtering cover of standard breeding cages [9]. Standard mice (St-1, n = 5; St-2, n = 7; St-3, n = 7; Fig. 1A) were reared under the same conditions except that the tea ball was left empty.

The deprived group (Fig. 5A) consisted of olfactory deprived animals (group D; n = 7; Fig. 5A). Animals were lightly anesthetized with a solution of ketamine (Ketavet; Gellini, Aprilia LT, Italy) and xylazine (Rompun; Bayer, Wuppertal, Germany) before inserting the nose plugs (polyethylene tubing, 0.7 mm) into the right naris for 42 days [42]. The effectiveness of olfactory deprivation was checked after sacrifice controlling that the nose plug was retrieved in the anterior part of the snout and confirming the reduced level of TH expression in the glomerular layer of the OB using immunohistochemistry [26]. Animals in which the plug was not retrieved or that did not show decreased TH expression were discarded.

### BrdU administration

To assess newborn cell survival, all mice of the experimental groups received four injections, 4 hours apart, of 5-bromo-2-deoxyuridine (BrdU; 50 mg/kg in 0.1 M Tris pH 7.4; Sigma, St. Louis, MO) on day 21 of the experiment and they were replaced in their respective cages for 10, 21 or 42 more days (Fig. 1A and Fig. 5A).

### Tissue preparation and sectioning

At the end of the survival time, mice were deeply anesthetized with an intraperitoneal injection of a solution of ketamine (Ketavet; Gellini, Aprilia LT, Italy) and xylazine (Rompun; Bayer, Wuppertal, Germany) and perfused transcardially with 0.9% saline, followed by 4% paraformaldehyde in 0.1 M phosphate buffer, pH 7.4. Brains were post fixed for 6 h in the same solution, cryoprotected in a 30% sucrose solution in 0.1 M phosphate buffer, pH 7.4, frozen, and cryostat sectioned (Leica). Free-floating coronal and serial sections (25 µm) were collected in multiwell dishes at the anatomical levels that comprised the entire OB. Sections were stored at −20°C in antifreeze solution until use.

### Immunohistochemistry

For BrdU immunostaining, sections were treated with 2 N HCl for 35 min at 37°C and neutralized with borate buffer, pH 8.5. Sections were incubated overnight at 4°C in primary antibodies diluted in 0.01 M PBS, pH 7.4, 0.5% Triton X-100, and 1% normal serum of the same species of the secondary antiserum. The primary antibodies used were anti-BrdU (1∶3000, rat, Oxford Biotechnology), anti-calbindin D-28K (CB) (1∶1000; rabbit; Swant, Bellinzona, Switzerland), anti-calretinin (CR) (1∶8000; rabbit; Swant), anti-tyrosine hydroxylase (TH) (1∶2000; rabbit; Institut Jacques Boy, Reims, France), anti-Glutamic acid decarboxylase-67 (GAD67) (1∶5000, monoclonal mouse, Chemicon, Temecula, CA), anti-polysialylated form of neural cell adhesion molecule (PSA-NCAM) (1∶2500; monoclonal mouse IgM; Abcys, Paris, France), anti-doublecortin (DCX) (1∶300; goat; Santa Cruz Biotechnology).

For double labeling with BrdU, sections were first incubated overnight at 4°C in anti-CB, anti-CR, anti-TH, anti-GAD67, anti-PSA-NCAM or anti-DCX primary antibodies and appropriate serum and incubated for 1 h at room temperature in secondary antibodies. Sections were then processed for BrdU detection following the protocol described above. Secondary antibodies used were as follows: anti-mouse, anti-rat and anti-goat Cy3 conjugated (1∶800; Jackson ImmunoResearch, West Grove, PA); anti-rabbit and anti-rat biotinylated (1∶250; Vector, Burlingame, CA) followed by avidin FITC incubation (1∶400; Jackson ImmunoResearch). Sections were mounted, air dried, and coverslipped in polyvinyl alcohol with diazabicyclo-octane (DABCO) as an anti-fading agent.

### Cell counting and statistical analysis

All cell counts were conducted blind with regards to the mouse status. Cell counts and image analysis were performed on a Nikon microscope coupled with a computer-assisted image analysis system (Neurolucida software, MicroBrightField, Colchester, VT) or on a Fluo-View 500 confocal microscope (Olympus Instruments, San Francisco,CA). Confocal image z-stacks were captured through the thickness of the slice at 1 µm optical steps. These image stacks were used for cell counting or assembled into extended focus photographs; brightness, color, and contrast balanced and assembled into panels with CorelDraw 11 (Corel, Ottawa, Ontario).

To estimate the volume of each layer, camera lucida drawings of sections (6–8 sections per animal, 300 µm inter-section intervals) through the entire MOB (from the anterior MOB to the anterior AOB) were made from low-magnification photographs. The boundaries between layers were estimated from changes in cell density in sections stained with the nuclear dye 4′,6-diamidino-2-phenylindole (DAPI). The area of each section and layer in the traces was automatically calculated by Neurolucida software and the total volume of the OB and layers estimated applying the Cavaleri method.

All BrdU-positive cells (6–8 sections per animal, 300 µm inter-section intervals) through the entire MOB were counted in the GL and GCL. Profile density of BrdU-positive cells (number of labeled profiles/µm^2^) was calculated and the total number of labeled cells was estimated using the formula: T = (NXV) t where V is the volume, N the profile density in the layer, and t the thickness of the sections. The numbers of positive profiles were then related to the volume of the region of interest to express data as a number of profiles per mm^3^ (volumetric density).

To establish the percentage of new-generated PGCs and GCs double-labelled for the different markers, serial sections from standard, enriched and deprived mice were examined systematically through the OB (3 sections positioned respectively at antero, medial and posterior level). To calculate the percentage of double-labelled cells for calbindin, calretinin and TH in the GL, all BrdU-positive cells were assessed. To calculate the percentage of double-labelled cells for BrdU and GAD67, PSA-NCAM or DCX in the GL and GCL a random sampling method was applied on confocal microscope. For each animal, the percentages of double-labelled cells into the GL and GCL were calculated over the total number of BrdU-positive cells counted. To calculate the total number of TH- positive cells in the GL, we applied a random sampling method on 3 sections (positioned respectively at antero, medial and posterior level) per animal; density and total number of TH- positive cells were calculated as described above. GAD67 signal intensity was measured by densitometry using ImageJ software (NIH, Bethesda, MD) on 3 sections (positioned respectively at antero, medial and posterior level) per animal and corrected to the background signal.

Statistical comparisons were conducted by Two-way ANOVA, with treatment (standard and enriched housing conditions) and survival time of newborn olfactory interneurons (10, 21 and 42 days) as independent variables. At each survival time Student's *t-*test or One-way ANOVA followed by Tukey post hoc comparison were performed where appropriate. Significance was established at P<0.05. All cell counts and volumes are presented as mean±standard error of the mean (SEM) and are derived from at least 3 different animals.
